# Persistent Horner Syndrome Following Benign Gynaecological Surgery: A Case Report and Review of the Literature

**DOI:** 10.7759/cureus.114170

**Published:** 2026-08-08

**Authors:** Melanie C Norton, Smriti Kuntal

**Affiliations:** 1 Obstetrics and Gynaecology, Lewisham and Greenwich NHS Trust, London, GBR; 2 Gynaecology, Lewisham and Greenwich NHS Trust, London, GBR

**Keywords:** hysterectomy, large fibroids, lower abdominal surgery, postoperative horner syndrome, ptosis

## Abstract

Horner syndrome is an uncommon neurological disorder resulting from the disruption of the oculosympathetic pathway and classically presents with ipsilateral ptosis, miosis and, variably, facial anhidrosis. Although recognised following cervical, thoracic and vascular procedures, it is rarely reported following benign gynaecological surgery. We present the case of a 44-year-old woman who developed persistent right-sided Horner syndrome following elective subtotal abdominal hysterectomy with bilateral salpingectomy performed under general anaesthesia with spinal anaesthesia. Right-sided ptosis developed on the first postoperative day and was followed by anisocoria without headache, neck pain, visual disturbance or diplopia. Horner syndrome was confirmed by apraclonidine testing. Extensive neurovascular investigations, including computed tomography (CT), CT angiography and magnetic resonance imaging (MRI), excluded intracranial, cervical and vascular pathology. Following multidisciplinary review, perioperative sympathetic chain injury related to neuraxial anaesthesia or patient positioning was considered the most likely explanation. Symptoms persisted at six months despite normal imaging. This case highlights the importance of recognising Horner syndrome after non-cervical surgery, promptly excluding life-threatening causes and considering perioperative sympathetic dysfunction as a diagnosis of exclusion.

## Introduction

Horner syndrome is an uncommon neurological disorder resulting from the interruption of the three-neuron oculosympathetic pathway extending from the hypothalamus to the eye. The classical clinical features include ipsilateral ptosis, miosis and variable facial anhidrosis, although the complete triad is not present in every patient. Recognition is clinically important because Horner syndrome may represent the first manifestation of potentially life-threatening pathology, including carotid artery dissection, brainstem infarction, cervical spinal cord lesions and apical lung malignancy [1-3]. Prompt recognition and appropriate investigation are therefore essential to exclude serious underlying disease before considering benign or iatrogenic causes.

Postoperative Horner syndrome has been described following cervical and thoracic surgery, internal jugular venous catheterisation and neuraxial anaesthesia. The majority of reported postoperative cases occur following obstetric epidural anaesthesia and are transient, resolving spontaneously within hours or days. Persistent Horner syndrome following non-cervical surgery is distinctly uncommon, and reports following benign gynaecological surgery are exceedingly rare [4-6].

We present the case of a woman who developed persistent Horner syndrome following elective subtotal abdominal hysterectomy with bilateral salpingectomy performed under combined general and spinal anaesthesia. Despite comprehensive neurovascular imaging, no structural or vascular cause was identified. In addition to describing the clinical course, we review the published literature on postoperative Horner syndrome and propose a practical diagnostic algorithm to facilitate the assessment of patients presenting with new-onset postoperative ptosis and anisocoria. 

## Case presentation

A 44-year-old multiparous woman was referred with symptomatic uterine leiomyomas causing heavy menstrual bleeding. Her medical history included bilateral avascular necrosis of both hips, one previous lower-segment Caesarean section and a laparoscopic appendicectomy. She was a non-smoker with no known neurological or ophthalmological disorders and worked as a teaching assistant.

Clinical examination demonstrated a fibroid uterus equivalent to approximately 14 weeks' gestation with obliteration of the pouch of Douglas. Preoperative pelvic imaging confirmed multiple posterior uterine leiomyomas. Following counselling regarding treatment options, she elected to undergo an elective subtotal abdominal hysterectomy with bilateral salpingectomy while preserving both ovaries.

The procedure was performed under combined general and spinal anaesthesia. The patient remained in the supine position throughout the operation. A Pfannenstiel incision was made through the previous Caesarean section scar. Adhesions between both ovaries and the posterior uterine fibroids were carefully divided before an uncomplicated subtotal abdominal hysterectomy with bilateral salpingectomy was completed. Both ovaries and the cervix were preserved. The procedure lasted approximately two hours. Estimated blood loss was 800 mL, of which 300 mL was reinfused using intraoperative cell salvage. The patient remained haemodynamically stable throughout the procedure. Histopathological examination confirmed benign uterine leiomyomas. Postoperatively, she received three doses of intravenous antibiotics and a 10-day course of prophylactic low-molecular-weight heparin before discharge.

**Figure 1:**
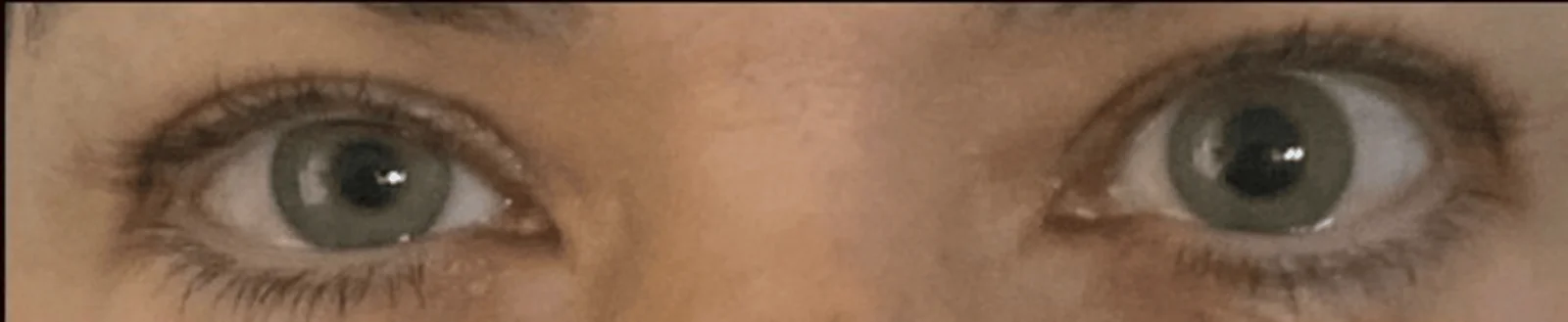
Visible right-sided ptosis with anisocoria

On the first postoperative day, the patient noticed drooping of the right upper eyelid. Over the following days, she developed anisocoria. She denied headache, neck pain, facial pain, visual disturbance, diplopia, dysphagia or other focal neurological symptoms. Clinical appearance following surgery is shown in Figure 1.

Ophthalmological assessment approximately two weeks after surgery demonstrated right-sided ptosis with ipsilateral miosis resulting in anisocoria. Apraclonidine (Iopidine) testing produced reversal of anisocoria with improvement in ptosis, confirming the diagnosis of Horner syndrome.

**Figure 2:**
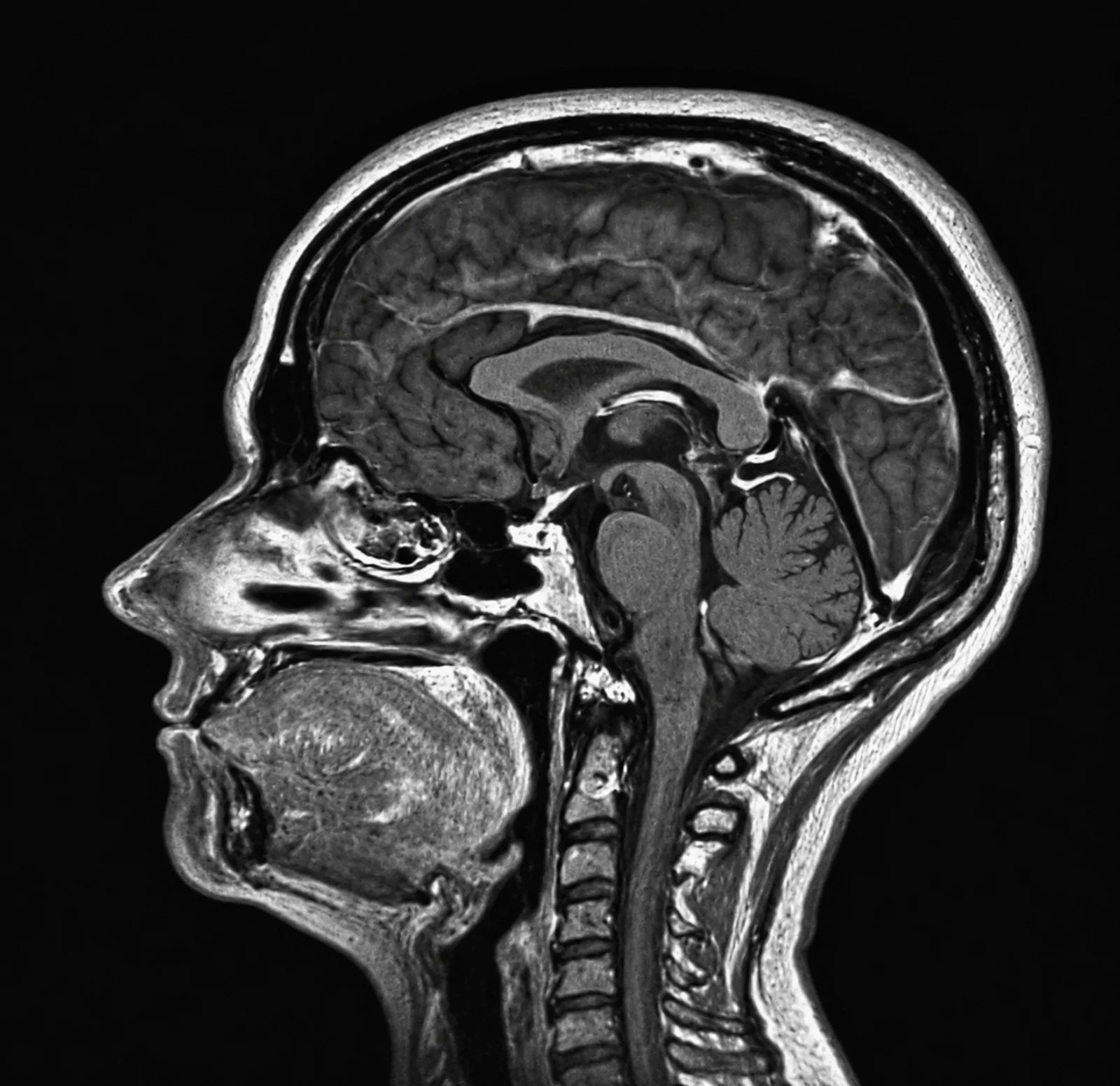
Magnetic resonance imaging of the head, sagittal view, which did not demonstrate intracranial pathology

A comprehensive neurovascular evaluation was undertaken. Computed tomography (CT) of the head demonstrated no acute intracranial abnormality. CT angiography of the intracranial circulation, carotid arteries and aortic arch excluded aneurysm, carotid artery dissection and other vascular pathology. Magnetic resonance imaging (MRI) of the brain, cervical spine and orbits demonstrated no structural abnormality. A mild kink of the right internal carotid artery at the C1 level was considered an incidental anatomical variant following neuroradiological review (Figure 2).

Routine postoperative blood investigations were unremarkable apart from expected postoperative anaemia. Haemoglobin was 104 g/L, white cell count 4.3×10⁹/L, neutrophils 2.8×10⁹/L, platelet count 320×10⁹/L and C-reactive protein <1 mg/L.

Following multidisciplinary review involving ophthalmology, neurology and neuroradiology, no structural or vascular cause for Horner syndrome was identified. In the absence of intracranial, cervical or vascular pathology, perioperative sympathetic dysfunction related to neuraxial anaesthesia or patient positioning was considered the most likely explanation.

At the six-month follow-up, the patient's symptoms had neither progressed nor resolved. Mild right-sided ptosis and ocular dryness persisted, although there was no deterioration in visual function.

Corrective eyelid surgery was discussed should the ptosis become functionally or cosmetically troublesome, and she was subsequently discharged from follow-up.

## Discussion

Horner syndrome is an uncommon but clinically important neurological syndrome resulting from the disruption of the three-neuron oculosympathetic pathway. Although the clinical findings of ipsilateral ptosis, miosis and facial anhidrosis are often subtle, prompt recognition is essential because Horner syndrome may be the presenting feature of serious underlying pathology, including carotid artery dissection, brainstem infarction, cervical spinal cord lesions and apical lung malignancy. Consequently, any patient presenting with new-onset Horner syndrome requires careful clinical assessment and appropriate neurovascular imaging before a benign or iatrogenic cause can be assumed [1-3].

The oculosympathetic pathway consists of a three-neuron chain extending from the hypothalamus to the eye. First-order neurons descend to the ciliospinal centre of Budge (C8-T2), second-order neurons ascend through the cervical sympathetic chain to the superior cervical ganglion, and third-order neurons travel along the internal carotid artery to the eye. Disruption at any point along this pathway results in the characteristic features of Horner syndrome [1-3].

In adults, the differential diagnosis is broad and includes central, preganglionic and postganglionic lesions affecting the sympathetic pathway. Common causes include cerebrovascular disease, carotid artery dissection, cervical spinal pathology, thoracic malignancy and iatrogenic injury following surgical or anaesthetic procedures. Sabbagh et al. demonstrated that although many cases of Horner syndrome are related to benign postoperative or traumatic causes, a significant proportion are attributable to vascular or neoplastic disease, emphasising the importance of urgent investigation, particularly in patients presenting with acute onset or associated pain [3]. The diagnostic imaging algorithm proposed by Davagnanam et al. recommends targeted vascular and cross-sectional imaging based on the clinical presentation, with particular emphasis on excluding carotid artery dissection because of its potentially catastrophic consequences [2].

Limitations were had, however, as serial objective measurements of eyelid position and pupillary responses were not available, limiting the assessment of subtle clinical progression over time.

**Figure 3:**
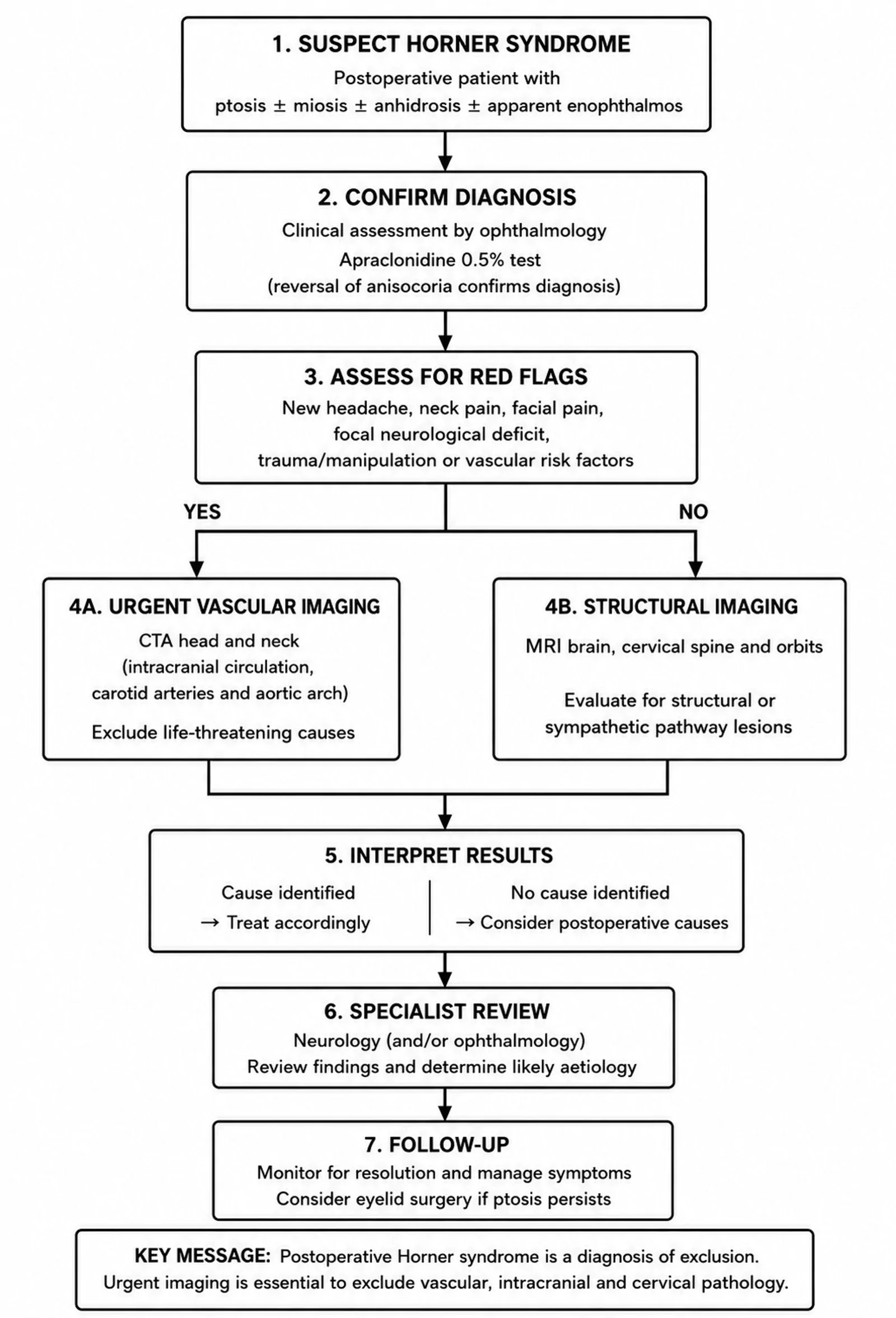
Diagnostic algorithm for postoperative Horner syndrome CTA: computed tomography angiography; MRI: magnetic resonance imaging

Our patient underwent a comprehensive diagnostic evaluation including CT of the head and CT angiography of the intracranial circulation, carotid arteries and aortic arch, together with MRI of the brain, cervical spine and orbits. These investigations excluded intracranial, cervical and vascular pathology, allowing perioperative sympathetic dysfunction to be considered as the most likely diagnosis. We believe that this systematic approach represents an important clinical learning point. Because postoperative ptosis may initially be attributed to local oedema, fatigue or residual anaesthetic effects, recognition of anisocoria and confirmation of Horner syndrome should prompt urgent investigation before perioperative causes are accepted. The diagnostic approach used in the present case is summarised in Figure 3.

Confirmation of Horner syndrome in our patient was achieved using apraclonidine testing. Apraclonidine has largely replaced cocaine testing in routine clinical practice because denervation hypersensitivity of the iris dilator muscle results in the reversal of anisocoria and improvement in ptosis, making it a highly sensitive and readily available diagnostic test in adults. The rapid availability of apraclonidine has facilitated the earlier confirmation of Horner syndrome and more timely investigation of potentially serious underlying pathology.

Postoperative Horner syndrome has most commonly been reported following neuraxial anaesthesia, particularly in obstetric patients receiving epidural analgesia. In their systematic review of 78 published cases, Chambers and Bhatia found that the majority of patients developed symptoms during or shortly after neuraxial blockade, with spontaneous resolution occurring within hours in most cases [4]. The proposed mechanism is the cephalad spread of local anaesthetic resulting in the temporary blockade of the preganglionic sympathetic fibres arising from the ciliospinal centre of Budge (C8-T2). Pregnancy-related anatomical and physiological changes, including engorgement of the epidural venous plexus and reduced epidural space volume, are thought to predispose obstetric patients to higher cephalad spread of local anaesthetic, explaining the predominance of cases within the obstetric literature.

Outside obstetrics, postoperative Horner syndrome is considerably less common. Isolated reports have described Horner syndrome following unintended subdural spread of local anaesthetic during epidural anaesthesia, prone-position surgery and thoracic spinal surgery [5-8]. Although the underlying mechanisms differ, these reports support the concept that interruption of sympathetic function may occur through neuraxial blockade, traction, compression or direct injury to the sympathetic chain. In contrast, our patient underwent uncomplicated benign abdominal surgery remote from the cervical sympathetic pathway, making direct surgical injury highly unlikely. Following multidisciplinary review by ophthalmology, neurology and neuroradiology, the most plausible explanation was perioperative sympathetic dysfunction related to either neuraxial anaesthesia or patient positioning, although the precise mechanism cannot be established.

An important feature distinguishing the present case from most previously published reports is the persistence of symptoms. Whereas the majority of reported postoperative cases resolve within hours or days, our patient continued to experience ptosis and ocular dryness six months after surgery. Hered et al. similarly described persistent Horner syndrome following thoracic spinal fusion and epidural analgesia, with prolonged symptoms ultimately requiring corrective eyelid surgery [6]. Persistent postoperative Horner syndrome therefore appears to represent a distinctly uncommon clinical outcome and may reflect more prolonged or severe disruption of sympathetic function than occurs in transient neuraxial blockade.

Although causality cannot be proven from a single case report, this case reinforces several important clinical principles. Firstly, new-onset postoperative ptosis should never be dismissed without careful examination of pupillary responses. Secondly, painful Horner syndrome or Horner syndrome associated with neurological symptoms should be regarded as a neurological emergency until carotid artery dissection and other life-threatening causes have been excluded. Finally, once structural pathology has been excluded, multidisciplinary assessment involving ophthalmology, neurology and neuroradiology is valuable in establishing the most likely aetiology and guiding follow-up.

The principal limitation of this report is that the exact mechanism of sympathetic pathway dysfunction cannot be definitively established. No investigation can reliably distinguish transient neuraxial sympathetic blockade from stretch-related injury or other perioperative mechanisms once structural pathology has been excluded. Furthermore, as with all single case reports, the findings cannot be generalised. Nevertheless, the rarity of persistent Horner syndrome following benign gynaecological surgery, together with the comprehensive diagnostic evaluation and prolonged follow-up presented here, provides a useful educational contribution to the existing literature (Table 1).

**Table 1:** Literature review of cases of Horner syndrome (postoperative)

Study	Surgical setting	Proposed mechanism	Time to onset	Outcome
Chambers and Bhatia (2018) [4]	Obstetric neuraxial blockade (systematic review of 78 cases)	Cephalad spread of local anaesthetic causing preganglionic sympathetic blockade	Usually during or within hours of neuraxial anaesthesia	Majority resolved spontaneously within hours (rarely >24 hours)
Rodríguez et al. (2005) [5]	Peripheral vascular surgery with epidural anaesthesia	Unintended subdural spread of local anaesthetic	Immediate postoperative period	Complete resolution within days
Hered et al. (1998) [6]	Thoracic spinal fusion with epidural analgesia	Sympathetic chain injury or prolonged neuraxial blockade	Immediate postoperative period	Persistent Horner syndrome requiring ptosis surgery

## Conclusions

Persistent Horner syndrome is an uncommon postoperative complication that may rarely occur following benign gynaecological surgery. This case demonstrates that even in the absence of headache, neck pain or focal neurological deficit, new-onset postoperative ptosis and anisocoria warrant urgent investigation to exclude carotid artery dissection, intracranial pathology and other serious causes before perioperative sympathetic dysfunction is considered.

Although the exact mechanism remains uncertain, neuraxial anaesthesia and perioperative positioning represent plausible explanations once structural pathology has been excluded. Increased awareness of this rare complication, together with a structured diagnostic approach and multidisciplinary management, may facilitate earlier diagnosis, appropriate reassurance and optimal patient care.
